# Curative gene therapies for rare diseases

**DOI:** 10.1007/s12687-020-00480-6

**Published:** 2020-08-15

**Authors:** Rocio Maldonado, Sami Jalil, Kirmo Wartiovaara

**Affiliations:** 1 Stem Cells and Metabolism Research Program, University of Helsinki, Helsinki, Finland; 2 Clinical Genetics, Helsinki University Hospital, Helsinki, Finland

**Keywords:** CRISPR-Cas9, Gene therapy, Genetic engineering

## Abstract

Diseases caused by alterations in the DNA can be overcome by providing the cells or tissues with a functional copy of the mutated gene. The most common form of gene therapy implies adding an extra genetic unit into the cell. However, new genome engineering techniques also allow the modification or correction of the existing allele, providing new possibilities, especially for dominant diseases. Gene therapies have been tested for 30 years in thousands of clinical trials, but presently, we have only three authorised gene therapy products for the treatment of inherited diseases in European Union. Here, we describe the gene therapy alternatives already on the market in the European Union and expand the scope to some clinical trials. Additionally, we discuss the ethical and regulatory issues raised by the development of these new kinds of therapies.

The progression of diseases resulting from genetic alterations can be stopped or reversed if the affected cells or tissues overcome the genetic failure. Gene therapy has been suggested as a possible treatment for inherited conditions since the 1970s (Friedmann and Roblin 1972; Terheggen et al. 1975), and the first official trial was initiated in the USA in 1990. The therapy consisted of a viral vector that delivered a functional copy of the adenosine deaminase (ADA) gene into the T cells of a severe combined immunodeficiency patient (Blaese et al. 1995; Culver et al. 1991). The success of this first clinical trial promoted numerous trials throughout the decade. The situation changed in 1999, when the University of Pennsylvania reported fatal systemic inflammatory response during an experimental gene therapy trial for ornithine transcarbamylase deficiency (Raper et al. 2003). Consequently, the development of the field markedly slowed down, until China approved a gene therapy trial for head and neck cancer in 2003 (Lang et al. 2003; Han et al. 2003; Raty, Pikkarainen, Wirth et al. 2008). Thereafter, the number of trials has soared. In February 2020, the total number of conducted or ongoing gene therapy clinical trials exceeds 4000 (Clinical trials NIH 2020). Extensive testing and development have yielded, however, only a handful of therapeutic products^1^. Additionally, most trials and half of the products are designed for somatic-alteration diseases, mainly cancer. This review describes the potentially curative gene therapy treatments for inherited diseases. We examine the authorised therapies in the European market and describe promising approaches in clinical trials for specific disease groups. We exclude protein replacement and oligonucleotide-based therapies, which are seldom curative; and allogeneic cell transplantation from genetically healthy donors, which can be curative, but is not classified as a bioengineered gene therapy.

Therapeutic applications in genetic diseases vary significantly, and the preferred methods for successful treatments are highly disease dependant*.* An in vivo therapy delivers a therapeutic vector in the form of DNA, RNA or a virus, while an ex vivo therapy consists of genetically modified cells or tissues (Gene Therapy Net 2020). The European Medicine Agency (EMA) webpage (European Medicines Agency 2015) displays the precise classifications and explains the regulatory processes for different categories of genetic therapies. The regulatory requirements of gene therapy products greatly influence the development of new treatments and affect the time lag between scientific breakthroughs and newly available medicines.

## Gene therapy strategies

All the market-authorised genetic treatments and most of the ongoing trials rely on the addition of a genetic element to the cells, including the necessary parts for expression. Coded as RNA or DNA, the gene transfer happens via chemical or physical techniques with variable efficiency and safety profiles but more frequently using a virus (Kamimura et al. 2011, Table 1). This ultimately leads to the production of the desired RNA and/or protein in the target cells.

**Table 1:** Commonly used viral vectors in gene therapy

Vector	Transfection capacity	Integration	Restrictions
Adenovirus	< 7.5 kb	None	Causes immune response, short-term expression
Adeno-associated virus (AAV)	< 4.5 kb	Low	Causes immune response
Alphavirus	< 7.5 kb	None	Short-term expression
Herpesvirus	> 30 kb	None	May cause immune response
Retrovirus	< 8 kb	High	Risk of insertional mutagenesis. Just infects dividing cells
Lentivirus	8–10 kb	High	Risk of insertional mutagenesis
Vaccinia virus	25 kb	None	Short-term expression

(Baldo et al. 2014; Ura et al. 2014; Hanna et al. 2017; Lundstrom 2018)

Genetic modification or editing means that the existing genetic code inside a living cell is altered. All the gene-editing methods guide an effector, including a nuclease, to a target site in the genome (What is Genome Editing? NIH 2019). After a successful targeting, the effector catalyses the desired modification by creating a cut in one or both strands of DNA, modifying it or replacing it with a synthetic template (Gene Therapy Net 2020). Although probably the most known editing method is CRISPR-Cas9, the older zinc finger nuclease (ZFN) technique is further in clinical trials, and others also exist (Table 2). The latest, 2019-published prime editing, is based on a reverse transcriptase enzyme ‘writing’ a new text into the DNA (Anzalone et al. 2019). So far, only the original publication has reported positive results applying this novel method. Different gene transfer and editing methods have brought us to a situation where we could write practically anything inside the cells, with almost endless options. The complexity of the genome sets the limitations (Jensen et al. 2017), and some modifications are technically easier to achieve than others.

**Table 2:**
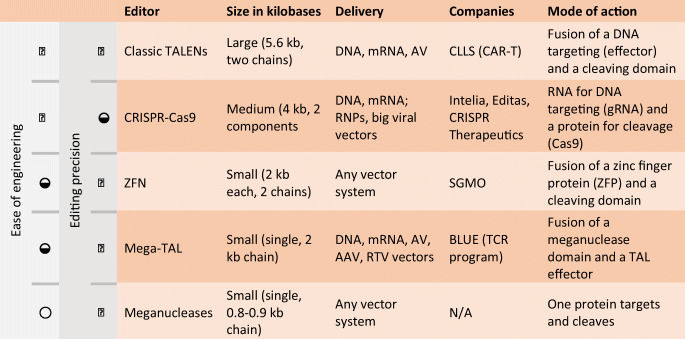
Available gene-editing tools^a^

^a^Modified from (BIOTECH Gene Therapy 2016)

*AV* Adenovirus, *AAV* adeno-associated virus, *RTV* retroviral, *RNP* ribonucleoprotein. **◯**, low; **◒,** moderate; **●,** high

## The cause of the disease defines the preferred techniques for a treatment

It is crucial to understand the pathogenesis of the target disease to appropriately design a gene therapy (Diakatou et al. 2019). A blood disease can be treated with a relatively small number of self-renewing haematopoietic stem cells (Boelens et al. 2013), but postmitotic cells—such as neurons—usually require a direct delivery of the therapeutic agent to a significant proportion of them (Naldini et al. 1996).

The exceptional potential of gene therapy was first envisioned in inherited diseases (Rosenberg et al. 1990), many of which lack appropriate therapies. Then, researchers extended the applications to acquired conditions. Some of the targets include infections, acquired ischemic and metabolic diseases and several types of cancer (Gene Therapy Net 2020). Yet, due to the large variety of mutations in cancerous cells (National Cancer Institute 2015), curative genetic therapies targeting malignant cells are difficult to develop. Therefore, therapies frequently aim to modify the genes in the cells that protect us from cancer (Eshhar et al. 1993; Maher et al. 2002; Zhang et al. 2017), although we will not describe them in detail.

**Fig. 1:**
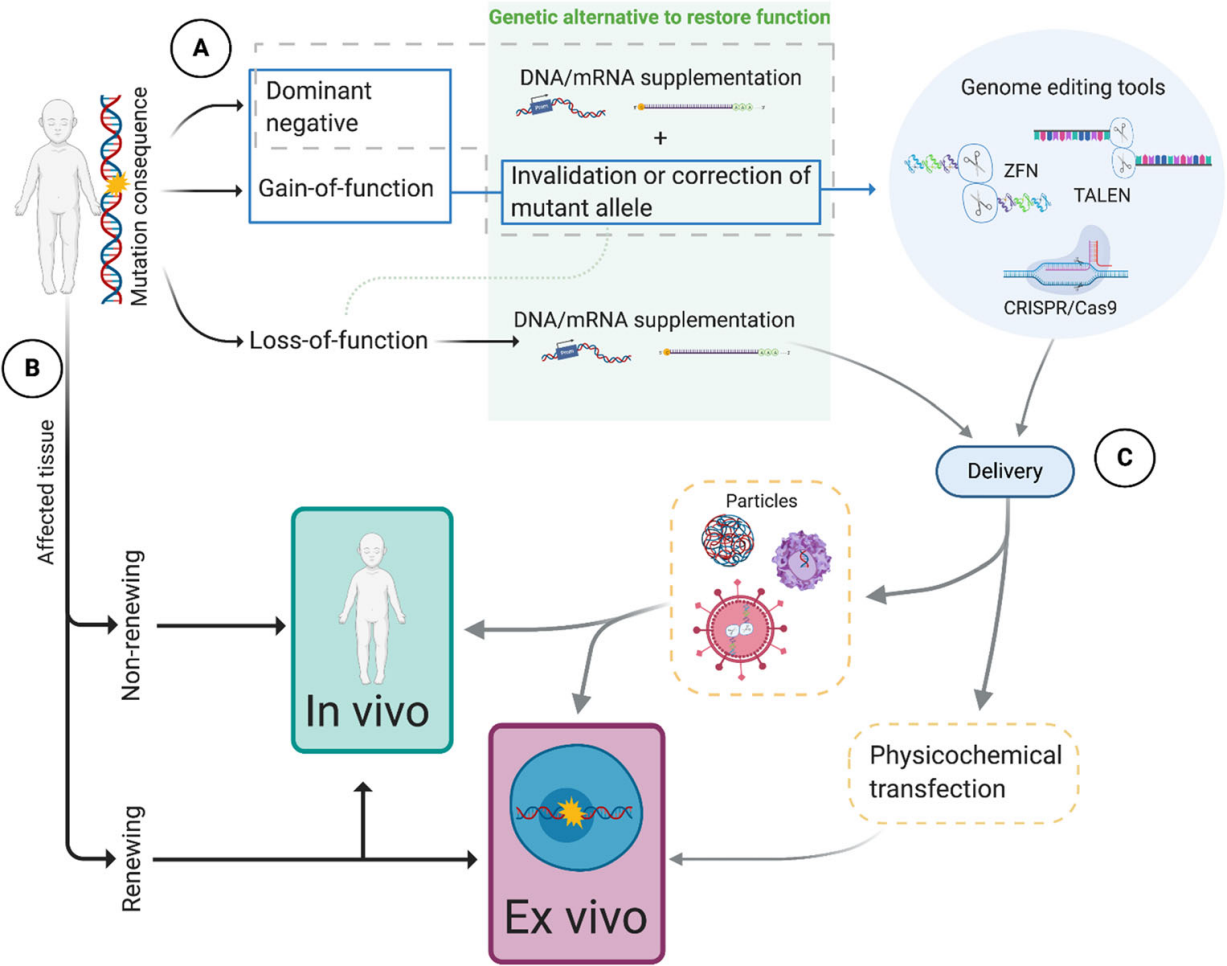
Flow chart model of biological and technical variables describing gene therapy strategies. (**a**) Type of disease mutation. Loss-of-function mutations can be treated by supplying the cells with a functional copy of the gene in the form of DNA or mRNA. If the disease-causing mutation results in gain-of-function or dominant-negative product, the current alternatives imply correcting the alteration or excising the altered allele using gene-editing tools (ZFN, TALEN, CRISPR/Cas9). (**b**) The affected tissue type has a major influence whether the disease can be targeted using in vivo or ex vivo therapies. Self-renewing tissues are much more approachable with ex vivo treatments. (**c**) Delivery options are determined by the tissue type and approach. Viral particles can be used both in vivo and ex vivo*.* Chemical or physical means are mainly used in ex vivo therapies. Created with BioRender.com

On top of understanding the cell and tissue pathogenesis of the disease, it is essential to know the molecular consequence of the disease mutation (Fig. 1). Recessive diseases are typically caused by loss-of-function mutations (Deutschbauer et al. 2005) and can be potentially cured by introducing a healthy copy of the gene into the cells. The same approach works for dominant diseases caused by haploinsufficiency (Hafler et al. 2016). On the contrary, if the pathogenicity raises from gain-of-function or dominant-negative gene products, gene/mRNA supplementation may not be sufficient. A common example of a gain-of-function mutation result is an overactive tyrosine kinase receptor, which cannot be silenced by the wild-type gene. Hence, in these cases, more suitable alternative for therapy would be to correct the mutation and/or excise the altered allele (Farrar et al. 2012; Mendes and Cheetham 2008).

## Clinical experience

In the EU, there have been seven market-authorised gene therapy products, six of which are still available (Table 3): three immunological gene therapies for cancer and three for treating inherited diseases. Of the latter, one is an in vivo viral therapy and two are ex vivo-modified cellular therapies. All these gene therapies add a new sequence to the target cell, and no agency has approved a gene-editing medicinal product yet. Despite the small number of current market authorisations, the technical improvements occur fast, and there are dozens of products in the regulatory process (European Medicines Agency 2020), suggesting gene therapy as a promising future field in medicine.

**Table 3:** Gene therapy products approved by the EMA^a^

Trade name	Product	Condition	Vector	EMA approval
Glybera®	Alipogene tiparvovec	Lipoprotein lipase deficiency		10/2012^†2017^
Imlygic®	Talimogene laherparepvec	Regionally or distantly metastatic unresectable melanoma	HSV-1/GM-CSF	12/2015
Strimvelis®^b^	Autologous CD34+ cells transduced to express ADA	Adenosine deaminase deficiency (ADA)	ϒ-retrovirus/ADA	05/2016
Kymriah® ^c^	Tisagenlecleucel	• Relapsed or refractory B-cell acute lymphoblastic leukaemia • Relapsed or refractory diffuse large B-cell lymphoma	LV-CAR (CD19R)	09/2018
Yescarta®^c^	Axicabtagene ciloleucel (CAR-T)	• Relapsed or refractory DLBCL and primary mediastinal large B-cell lymphoma • Some types of non-Hodgkin lymphoma	ϒ-retrovirus	08/2018
LUXTURNA®^d^	Voretigene neparvovec	Inherited retinal dystrophy caused by biallelic *RPE65* mutations	AAV2-RPE65	11/2018
Zynteglo®^e^	Autologous CD34+ cells encoding βA-T87Q-globin gene	β-thalassemia with regular blood transfusions	LV-ß-globin	05/2019

†Taken out of the market

^a^Gene Therapy Net (2020)

^b^Novartis (2020)

^c^Dolgin (2019)

^d^Master (2019)

^e^DBGen (2019)

^f^Deena Beasley (2019)

The first European market authorisation for a gene therapy product was given in 2012 to Glybera® but was later suspended for commercial reasons (European Medicines Agency 2017). The second approved product was the haematological cell product Strimvelis®, for the treatment of the ‘bubble boy‘-immune deficiency ADA-SCID (European Medicines Agency 2016). The therapy involves the retroviral addition of the gene to the patient cells to surpass the insufficiency of the X-linked ADA gene causing the disease.

The only available in vivo gene therapy product is the adeno-associated virus (AAV)-based LUXTURNA® (FDA 2017; European Medicines Agency 2019a, b). It is administered as a subretinal injection in patients with biallelic *RPE65* gene mutations, suffering from Leber’s congenital amaurosis (eye disease, NCT00999609).

The latest gene therapy product arrived in the market in 2019, when the EMA approved Zynteglo® (European Medicines Agency 2019a, b). The product consists of autologous haematopoietic stem cells treated ex vivo with a lentivirus to express a functional β-globin gene in patients suffering from thalassemia (NCT01745120).

The databases of the EU Clinical Trial Register (2020), National Institute of Health (Clinical trials NIH 2020) and the World Health Organization (2020) better illustrate the fast development and current situation of gene therapy trials. In total, thousands of trials have been registered, including more than 300 phase 3 gene therapy studies. Next, we describe gene therapy products and some ongoing clinical trials for inherited haematological, ophthalmological and metabolic diseases (Table 4).

**Table 4:** Examples of gene therapy clinical trials for inherited haematological, ophthalmological and metabolic diseases

	Condition	Target	Clinical trial ID	Method		Delivery
Blood	B-thalassemia	*BCL11a*	NCT03432364	ZFN	Ex vivo	Non-viral
			NCT03653247		Ex vivo	N.R.
			NCT03655678	CRISPR-Cas9	Ex vivo	Non-viral
			NCT03745287		Ex vivo	Non-viral
		β-globin	NCT03728322	CRISPR-Cas9	iHSC	N.R.
		βA-T87Q-globin	**NCT01745120 (Zynteglo)**	Gene suppl.	Ex vivo	LV
Eye	LCA	*RPE65*	NCT00643747	Gene suppl.	In vivo	AAV2
			NCT00481546		In vivo	AAV2
			NCT00821340		In vivo	AAV2
			**NCT00999609 (Luxturna)**		In vivo	AAV2
			NCT03872479	CRISPR-Cas9	In vivo	AAV5
	LHON	*ND4*	NCT03428178	Gene suppl	In vivo	AAV2
			NCT03153293		In vivo	AAV2
			NCT02064569		In vivo	AAV2
			NCT02161380		In vivo	AAV2
	Achromatopsia	*CNGA3*	NCT02935517	Gene suppl	In vivo	AAV2
		*CNGA3/CNGB3*	NCT03278873	Gene suppl	In vivo	AAV2/8
	Choroideremia	*REP1*	NCT02341807	Gene suppl	In vivo	AAV2
			NCT03507686		In vivo	AAV2
Metabolism	PKD	*RPK* (Red cell PK)	NCT04105166	Gene suppl	Ex vivo	LV
	Haemophilia	*F8*	NCT03061201	Gene suppl	In vivo	AAV2/6
	ADA-SCID	*ADA*	**NCT01380990 (Strimvelis)**	Gene suppl	Ex vivo	LV
	Fabry disease	*GLA*	NCT04046224	Gene suppl	In vivo	AAV2/6
	MPS type I	*IDUA*	NCT02702115	ZFN + gene suppl	In vivo	AAV2/6
			NCT03488394	Gene suppl	Ex vivo	LV
	MPS type II	*IDS*	NCT03041324	ZFN + gene suppl	In vivo	AAV2/6

*N.R.* Not reported. Bold: EMA-approved products

### Haematopoietic stem and progenitor cells

Haematopoietic stem and progenitor cells (HSPCs) are a particularly promising cell population for gene therapies due to their relatively easy extraction and reintroduction into the patient and their well-described behaviour (Juric et al. 2016). This cell population survives ex vivo manipulation and transplantation into the same subject (autologous transplantation) or into another recipient (allogeneic transplantation) (Juric et al. 2016). They present a remarkably positive response to many cell engineering approaches, namely, ex vivo electroporation of ribonucleoprotein and mRNA or transduction with lentivirus (LV) and AAV (Bjurström et al. 2016; Hendel et al. 2015; Roselli et al. 2010). Additionally, according to the EMA, an estimated 36,000 patients a year receive HSPC transplantation in the EU. Thus, this well-tested procedure does not represent a major concern for ex vivo gene therapy clinical trials targeting the HSPC population. Therefore, the most efficient gene-editing and gene transfer clinical trials involve ex vivo strategies. However, performing HSPC-based therapies on a big scale still represents a challenge for hospital infrastructure and reproducible manufacturing (Bai et al. 2019).

As of February 2020, 48 phases 1 or 2 clinical trials for genetic therapies in HSPC have been approved (Clinical trials NIH 2020). From these, 43 propose gene transfer methods, introducing a functional cDNA into the patient’s HSPC. The cDNA product, regulated by a stable promotor, replaces the missing or dysfunctional protein. To deliver expression cassette, all these trials utilise retroviruses, including LV and self-inactivating gammaretrovirus. An example of this approach is the recently approved Zynteglo®(NCT01745120) that uses lentiviral delivery of a working βA(T87Q)-globin gene sequence. In theory, AAVs could also be employed to deliver a stable cDNA expression cassette as shown in other clinical trials targeting different tissues (Dunbar et al. 2018).

The remaining 5 clinical trials correspond to gene-editing approaches. Four of them aim to disrupt the erythroid enhancer of the BCL11a gene, which induces the expression of γ-globin while decreasing the β-globin (Psatha et al. 2018). Of these, two are based on the introduction of ZFN mRNA, and the other two employ non-viral delivery of CRISPR-Cas9.

Finally, there is just one clinical trial aiming to correct the β-thalassemia mutations in the β-globin gene, targeting induced haematopoietic stem cells (iHSC) instead of HSPC. Working with iHSC allows clonal selection or population enrichment of the edited cells, resulting in a more controlled and standardised product. Nevertheless, iHSC transplantation is not approved as a therapy yet due to concerns about its functionality and safety (Tan et al. 2018). Hence, before gene therapies in iHSC become a reality, it remains necessary to test and further characterise these engineered cells for human transplantation.

### Eye diseases

Gene therapies for eye diseases have been widely explored and represent promising alternatives for several conditions, such as Leber’s congenital amaurosis (LCA), Leber’s hereditary optic neuropathy (LHON), achromatopsia and choroideremia, among others.

Viral vectors are currently the chosen mean to deliver functional copies of genes. Already in 2008, three studies disclosed the successful treatment of LCA (Bainbridge et al. 2008; Cideciyan et al. 2008; Maguire et al. 2008). In all of them, the patients received a subretinal injection of AAVs carrying a functional copy of the *RPE65* gene. Regarding LHON, for which the current treatments involve oestrogen replacement (Fantini et al. 2019; Giordano et al. 2011; Giordano et al. 2014) or administration of idebenone (Mashima et al. 2000), the gene therapies in clinical trials aim to become a sustained solution for the condition. For conditions for which the existing treatment only delays the progression of the symptoms, genetic approaches could offer to stop or reverse it. Such are the cases of choroideremia and congenital achromatopsia, currently aided with diet management (Kalatzis et al. 2013; Patrício et al. 2018) and eyeborg (Rochi 2009), respectively.

Several genetic therapies in clinical trials have yielded promising results. Yet, it was not until late 2017 that the Food and Drug Administration (FDA) approved LUXTURNA®, the first in vivo gene therapy product for *RPE65*-caused LCA (FDA 2017). More recently, Editas Medicine and Allergan started the first clinical trial for an in vivo CRISPR-based gene therapy in humans (NCT03872479; Editas Medicine, Allergan 2019, 2020). The study tests the effects of AGN-151587 (EDIT-101) in *CEP290* gene, administering a single dose via subretinal injection. The aim is to deliver gene-editing tools directly into the affected cells in patients with LCA10, where they would correct the disease-causing mutation.

### Inborn errors of metabolism

Inherited metabolic diseases are generally caused by genetic mutations affecting enzyme expression or function, resulting in metabolism impairment (MeSH 2020; MedlinePlus n.d.). The diverse pathogenesis and the wide spectrum of phenotypes demand an equally wide, albeit specific, range of treatments.

Traditionally, some of these diseases were approached with dietary modulation (restriction or supplementation). However, many others remained untreatable until enzyme replacement, organ transplantation and gene therapy became common, around 20 years ago (Fukao and Nakamura 2019). Currently, several genetic therapies in clinical trial target metabolic diseases, including Pyruvate Kinase Deficiency (PKD), haemophilia, ADA-SCID, Fabry disease and mucopolysaccharidosis (MPS) type I and II (also known as Hunter syndrome). Although all of them employ viral vectors to deliver functional copies of the patients’ dysfunctional genes, Sangamo’s products (NCT02702115, NCT03041324) present the first in vivo gene-editing approaches. Recombinant AAV2/6 introduce ZFNs and a correct copy of α-L-iduronidase or iduronate 2-sulfatase for MPS I or II, respectively. These genes are placed under the control of the highly active albumin promoter in the patients’ own hepatocytes (Laoharawee et al. 2018). Hence, they do not require a massive infection efficiency, while they provide a permanent, tissue-specific expression of the desired gene.

Another trial by the same company adopts a similar workflow for haemophilia A. The SB-525 vector encodes the cDNA for the β-domain of the human clotting factor VIII (hF8) under a liver-specific promoter. The aim is to establish a stable and long-term secretion of F8 after a single administration of the AAV2/6 product. Thus, this gene therapy protects the patients against bleeding while freeing them from recurrent F8 replacement treatments.

## Regulation and ethics

Each member state of the EU manages the authorisation of clinical trials, but EMA is the entity that ensures that the quality, safety and ethical aspects of the therapy comply with the EU legislation. The assessment of therapies’ safety and efficacy requires an understanding in molecular and cell biology, and the classification based on the mechanism of actions is not always easy. Within the EMA, the regulation of genetic medical products is administered by the Committee for Advanced Therapies (CAT), under the classification of advanced therapy medicinal products (ATMP)(European Medicines Agency (n.d.)).

Gene therapy shares many general concerns with other traditional therapies: the risk-benefit ratio must be acceptable, and the same patient-rights issues are valid (National Academy of Science 2017). However, gene therapy does present some specific concerns. One of the most debated points is the potential of germline transmission. The Oviedo Convention by the Council of Europe prohibits inherited changes (Council of Europe 1997). Additionally, the scientific consensus is that we do not yet have enough experience in somatic therapies and techniques to safely consider germline therapies (National Academy of Science 2017). Nevertheless, entities like the Nuffield Council on Bioethics have argued that it would be unethical not to treat if a treatment is available (Nuffield Council on Bioethics 2018). The germline transmission of a healthy copy of a gene offers potentially stopping the further inheritance of pathogenic mutations altogether. In this regard, the fast development of gene therapies and the improvement of their accuracy and safety may change the regulatory situation.

Furthermore, novel economic and social concerns emerged with gene therapies and other new treatments for rare diseases. Occasionally, the small number of patients turns impossible to reach the demands of the conventional pharmacological drug development, which affects the commercial interest of the industry. Moreover, some diseases are only approachable with genetic treatments, giving rise to financial toxicity (Zafar and Abernethy 2013). This means that the few available manufacturers can price their products as high as possible. The argument of commercial partners often relies on the substantial price-outcome differences between their product and the existing therapies (Green 2019). In Europe, the centralised health care systems balance cost-effectiveness analyses often in favour of the therapy, as the long-term health benefits of the low number of patients justifies the investment. Yet, some years ago, financial toxicity influenced greatly Glybera’s market authorization withdrawal (European Medicines Agency 2017) and placed the pricing of novel therapies as an issue for discussion. Nowadays, the prices of the EMA-approved gene therapy products (Table 3) range from €28 thousand/year (Imlygic) to €1.575 million (Zynteglo).

## Concluding remarks

The emerging interest for gene therapy began in the 1990s, and, in hindsight, the techniques and regulation were insufficient at the time, leading to some serious adverse effects and even deaths. In the last years, these setbacks have been scarce, if any, which has brought back the enthusiasm and increased the funding. The optimism is encouraged by gene-editing and other novel or improved techniques, the number of which seems to increase every week (Mitha 2019). While gene-editing approaches remain in early phases, the follow-up times in many gene therapy trials have exceeded the 10 years without major undesired effects reported to ClinicalTrials.

Gene therapies may provide possible curative treatments for genetic diseases. However, further understanding of each disease’s cellular and genetic pathology is needed. Technical improvements are also required (specifically to deliver products to non-dividing cells), as well as making them specific to increase safety. Developing new treatments always bears risks, but if the potential benefit proves significant, some risks are acceptable. The regulatory questions seem solvable and have not hindered critical development. The question of germline modifications has made many headlines but seems so far to be quite marginal in practical terms. However, it is still an important unresolved issue but outside the scope of this review.

Additional challenges remain unmet. In 2017, the Massachusetts Institute of Technology predicted that by 2022, the FDA would have approved almost 40 new gene therapies (MIT NEWDIGS Initiative 2017). Europe could expect a similar situation. This may require changes in existing social and economic structures if we aim to include these advance therapies as regular medical practices. Scientists, doctors and governments ought to focus on informing and preparing the society to assimilate the arising novel treatments. Health care systems need the adequate hospital infrastructure and educated professionals to ensure competence and applicability.

While scientific research explores treatments for a wide spectrum of rare diseases, financial toxicity still threatens accessibility and availability. Thus, the new therapies must result cost-efficient enough to succeed and remain in the market. Moreover, in countries with limited health care support, these expenses become an additional burden for economically vulnerable patients. Assuring accessibility challenges societies and the scientific community aiming to ensure the equal treatment of the citizens.

In summary, advances in scientific research and gene therapy clinical trials promise great advantages in the long-term treatment of diseases, including rare genetic conditions. The field progresses rapidly, and varied approaches are explored and/or already in clinical trials. In the foreseeable future, societies and international agencies may need to re-evaluate and update the current regulations in accordance with therapy development. Further improvements and adaptations require collaborative efforts of multidisciplinary teams (including governments) to make the breakthroughs accessible for everyone.

## Footnotes

1 In this review, the term *therapeutic product* refers to those therapies that have been approved for market commercialisation.

## References

[CR1] Anzalone AV, Randolph PB, Davis JR, Sousa AA, Koblan LW, Levy JM, Chen PJ, Wilson C, Newby GA, Raguram A, Liu DR (2019). Search-and-replace genome editing without double-strand breaks or donor DNA. Nature..

[CR2] Bai T, Li J, Sinclair A, Imren S, Merriam F, Sun F, O’Kelly MB, Nourigat C, Jain P, Delrow JJ, Basom RS, Hung H-C, Zhang P, Li B, Heimfeld S, Jiang S, Delaney C (2019) Expansion of primitive human hematopoietic stem cells by culture in a zwitterionic hydrogel. Nat Med 25(10):1566–1575 10.1038/s41591-019-0601-5 31591594

[CR3] Bainbridge JWB, Smith AJ, Barker SS, Robbie S, Henderson R, Balaggan K, Viswanathan A, Holder GE, Stockman A, Tyler N, Petersen-Jones S, Bhattacharya SS, Thrasher AJ, Fitzke FW, Carter BJ, Rubin GS, Moore AT, Ali RR (2008). Effect of gene therapy on visual function in Leber’s congenital amaurosis. N Engl J Med.

[CR4] Baldo A, den Akker E, Bergmans H, Lim F, Pauwels K (2014). General considerations on the biosafety of virus-derived vectors used in gene therapy and vaccination. Curr Gene Ther.

[CR5] BIOTECH Gene Therapy (2016) https://twitter.com/_b_i_o_t_e_c_h_/status/774586084575551488. Accessed 17 February 2020

[CR6] Bjurström CF, Mojadidi M, Phillips J, Kuo C, Lai S, Lill GR, Cooper A, Kaufman M, Urbinati F, Wang X, Hollis RP, Kohn DB (2016) Reactivating Fetal Hemoglobin Expression in Human Adult Erythroblasts Through BCL11A Knockdown Using Targeted Endonucleases. Mol Ther - Nucleic Acids 5:e351 10.1038/mtna.2016.52 PMC5023398 28131278

[CR7] Blaese RM, Culver KW, Miller AD, Carter CS, Fleisher T, Clerici M, Shearer G, Chang L, Chiang Y, Tolstoshev P, Greenblatt JJ, Rosenberg SA, Klein H, Berger M, Mullen CA, Ramsey WJ, Muul L, Morgan RA, Anderson WF (1995). T lymphocyte-directed gene therapy for ADA-SCID: initial trial results after 4 years. Science..

[CR8] Boelens JJ, Aldenhoven M, Purtill D, Ruggeri A, DeFor T, Wynn R (2013). Outcomes of transplantation using various hematopoietic cell sources in children with Hurler syndrome after myeloablative conditioning. Blood..

[CR9] Cideciyan AV, Aleman TS, Boye SL, Schwartz SB, Kaushal S, Roman AJ, Pang JJ, Sumaroka A, Windsor EAM, Wilson JM, Flotte TR, Fishman GA, Heon E, Stone EM, Byrne BJ, Jacobson SG, Hauswirth WW (2008). Human gene therapy for RPE65 isomerase deficiency activates the retinoid cycle of vision but with slow rod kinetics. Proc Natl Acad Sci U S A.

[CR10] Clinical trials NIH (2020) https://clinicaltrials.gov/. Accessed February 2020

[CR11] Council of Europe (1997) Convention for the protection of human rights and dignity of the human being with regard to the application of Biology and Medicine: convention on human rights and biomedicine. Council of Europe. https://rm.coe.int/CoERMPublicCommonSearchServices/DisplayDCTMContent?documentId=090000168007cf98. Accessed 20 February 2020

[CR12] Culver KW, Osborne WRA, Miller AD, Fleisher TA, Berger M, Anderson WF, Blaese RM (1991) Correction of ADA deficiency in human T lymphocytes using retroviral-mediated gene transfer. Transplant Proc 1846711

[CR13] DBGen (2019) Gene therapies open very promising scenarios for the treatment of genetic blindness. DBGen Ocular Genetics. https://dbgen.com/en/gene-therapies-open-very-promising-scenarios-for-the-treatment-genetic-blindness/. Accessed 20 February 2020

[CR14] Deena Beasley TM (2019) Bluebird prices gene therapy at 1.58 million euros over 5 years. REUTERS Health News. https://www.reuters.com/article/us-bluebird-bio-gene-therapy-price/bluebird-prices-gene-therapy-at-1-575-million-euros-over-five-years. Accessed 20 February 2020

[CR15] Deutschbauer AM, Jaramillo DF, Proctor M, Kumm J, Hillenmeyer ME, Davis RW, Nislow C, Giaever G (2005). Mechanisms of haploinsufficiency revealed by genome-wide profiling in yeast. Genetics..

[CR16] Diakatou M, Manes G, Bocquet B, Meunier I, Kalatzis V (2019) Genome editing as a treatment for the most prevalent causative genes of autosomal dominant retinitis pigmentosa. Int J Mol Sci 20. 10.3390/ijms20102542 10.3390/ijms20102542 PMC6567127 31126147

[CR17] Dolgin E (2019). “Bubble boy” gene therapy reignites commercial interest. Nat Biotechnol.

[CR18] Dunbar CE, High KA, Joung JK, Kohn DB, Ozawa K, Sadelain M (2018) Gene therapy comes of age. Science 359(6372):eaan4672 10.1126/science.aan4672 29326244

[CR19] Editas Medicine, Allergan (2019). Allergan and Editas Medicine initiate the brilliance phase 1/2 clinical trial of AGN-151587 (EDIT-101) for the treatment of LCA10.

[CR20] Editas Medicine, Allergan (2020). Allergan and Editas Medicine announce dosing of first patient in Landmark Phase 1/2 Clinical Trial of CRISPR Medicine AGN-151587 (EDIT-101) for the Treatment of LCA10.

[CR21] Eshhar Z, Waks T, Gross G, Schindler DG (1993). Specific activation and targeting of cytotoxic lymphocytes through chimeric single chains consisting of antibody-binding domains and the γ or ζ subunits of the immunoglobulin and T-cell receptors. Proc Natl Acad Sci U S A.

[CR22] EU Clinical Trial Register (2020) www.clinicaltrialsregister.eu. Accessed February 2020

[CR23] European Medicines Agency (2015) Reflection paper on classification of advanced therapy medicinal products: https://www.ema.europa.eu/en/documents/scientific-guideline/reflection-paper-classification-advanced-therapy-medicinal-products_en-0.pdf. Accessed 22 February 2020

[CR24] European Medicines Agency (2016) Strimvelis EPAR*.* European Medicines Agency. https://www.ema.europa.eu/en/documents/product-information/strimvelis-epar-product-information_en.pdf. Accessed 02 February 2020

[CR25] European Medicines Agency. (2017) Glybera EPAR*.* European Medicines Agency. https://www.ema.europa.eu/en/documents/product-information/glybera-epar-product-information_en.pdf. Accessed 02 February 2020

[CR26] European Medicines Agency (2019a) Luxturna EPAR*.* European Medicines Agency. https://www.ema.europa.eu/en/documents/product-information/luxturna-epar-product-information_en.pdf. Accessed 02 February 2020

[CR27] European Medicines Agency (2019b) Zynteglo EPAR*.* European Medicines Agency. https://www.ema.europa.eu/en/documents/overview/zynteglo-epar-medicine-overview_en.pdf. Accessed 02 February 2020

[CR28] European Medicines Agency (2020). Applications for new human medicines under evaluation by the Committee for Medicinal Products for Human Use.

[CR29] European Medicines Agency (n.d.) Advanced therapy classification. European Medicines Agency. https://www.ema.europa.eu/en/human-regulatory/marketing-authorisation/advanced-therapies/advanced-therapy-classification. Accessed 05 February 2020

[CR30] Fantini M, Asanad S, Karanjia R, Sadun A (2019). Hormone replacement therapy in Leber’s hereditary optic neuropathy: accelerated visual recovery in vivo. J Curr Ophthalmol.

[CR31] Farrar GJ, Millington-Ward S, Chadderton N, Humphries P, Kenna PF (2012). Gene-based therapies for dominantly inherited retinopathies. Gene Ther.

[CR32] FDA (2017) Luxturna Approval Letter*.* U.S Food & Drug Administration: https://www.fda.gov/media/109487/download. Accessed 02 February 2020

[CR33] Friedmann T, Roblin R (1972). Gene therapy for human genetic disease?. Science..

[CR34] Fukao T, Nakamura K (2019). Advances in inborn errors of metabolism. J Hum Genet.

[CR35] Gene Therapy Net (2020) http://www.genetherapynet.com/types-of-gene-therapy.html. Accessed February 2020

[CR36] Giordano C, Montopoli M, Perli E, Orlandi M, Fantin M, Ross-Cisneros FN, Caparrotta L, Martinuzzi A, Ragazzi E, Ghelli A, Sadun AA, d’Amati G, Carelli V (2011). Oestrogens ameliorate mitochondrial dysfunction in Leber’s hereditary optic neuropathy. Brain..

[CR37] Giordano C, Iommarini L, Giordano L, Maresca A, Pisano A, Valentino ML, Caporali L, Liguori R, Deceglie S, Roberti M, Fanelli F, Fracasso F, Ross-Cisneros FN, D’Adamo P, Hudson G, Pyle A, Yu-Wai-Man P, Chinnery PF, Zeviani M, Salomao SR, Berezovsky A, Belfort R, Ventura DF, Moraes M, Moraes Filho M, Barboni P, Sadun F, de Negri A, Sadun AA, Tancredi A, Mancini M, d’Amati G, Loguercio Polosa P, Cantatore P, Carelli V (2014). Efficient mitochondrial biogenesis drives incomplete penetrance in Leber’s hereditary optic neuropathy. Brain..

[CR38] Green A (2019) Biotech companies defend prices of one-off gene therapy. Financial Times Online, pp. https://www.ft.com/content/edd639fc-9755-11e9-98b9-e38c177b152f. Accessed 15 February 2020

[CR39] Hafler BP, Comander J, Difranco CW, Place EM, Pierce EA (2016). Course of ocular function in PRPF31 retinitis pigmentosa. Semin Ophthalmol.

[CR40] Han Dm et al. (2003) Effectiveness of recombinant adenovirus p53 injection on laryngeal cancer: phase I clinical trial and follow up. Zhonghua Yi Xue Za Zhi 14703409

[CR41] Hanna E, Rémuzat C, Auquier P, Toumi M (2017) Gene therapies development: slow progress and promising prospect. J Market Access Health Policy 5. 10.1080/20016689.2017.1265293 10.1080/20016689.2017.1265293 PMC5328344 28265348

[CR42] Hendel A, Bak RO, Clark JT, Kennedy AB, Ryan DE, Roy S, Steinfeld I, Lunstad BD, Kaiser RJ, Wilkens AB, Bacchetta R, Tsalenko A, Dellinger D, Bruhn L, Porteus MH (2015) Chemically modified guide RNAs enhance CRISPR-Cas genome editing in human primary cells. Nat Biotechnol 33(9):985–989 10.1038/nbt.3290 PMC4729442 26121415

[CR43] Jensen KT, Fløe L, Petersen TS, Huang J, Xu F, Bolund L, Luo Y, Lin L (2017). Chromatin accessibility and guide sequence secondary structure affect CRISPR-Cas9 gene editing efficiency. FEBS Lett.

[CR44] Juric MK, Ghimire S, Ogonek J, Weissinger EM, Holler E, van Rood JJ, Oudshoorn M, Dickinson A, Greinix HT (2016) Milestones of Hematopoietic Stem Cell Transplantation – From First Human Studies to Current Developments. Front Immunol 7 10.3389/fimmu.2016.00470 PMC5101209 27881982

[CR45] Kalatzis V, Hamel CP, Macdonald IM (2013). Choroideremia: towards a therapy. Am J Ophthalmol.

[CR46] Kamimura K, Suda T, Zhang G, Liu D (2011). Advances in gene delivery systems. Pharmaceut Med.

[CR47] Lang FF, Bruner JM, Fuller GN, Aldape K, Prados MD, Chang S, Berger MS, McDermott MW, Kunwar SM, Junck LR, Chandler W, Zwiebel JA, Kaplan RS, Yung WKA (2003) Phase I Trial of Adenovirus-Mediated p53 Gene Therapy for Recurrent Glioma: Biological and Clinical Results. J Clin Oncol 21(13):2508–2518 10.1200/JCO.2003.21.13.2508 12839017

[CR48] Laoharawee K, DeKelver RC, Podetz-Pedersen KM, Rohde M, Sproul S, Nguyen HO, Nguyen T, St. Martin SJ, Ou L, Tom S, Radeke R, Meyer KE, Holmes MC, Whitley CB, Wechsler T, McIvor RS (2018). Dose-dependent prevention of metabolic and neurologic disease in murine MPS II by ZFN-mediated in vivo genome editing. Mol Ther.

[CR49] Lundstrom K (2018) Viral vectors in gene therapy. Diseases 6. 10.3390/diseases6020042 10.3390/diseases6020042 PMC6023384 29883422

[CR50] Maguire AM, Simonelli F, Pierce EA, Pugh EN, Mingozzi F, Bennicelli J (2008). Safety and efficacy of gene transfer for Leber’s congenital amaurosis. N Engl J Med.

[CR51] Maher J, Brentjens RJ, Gunset G, Rivière I, Sadelain M (2002). Human T-lymphocyte cytotoxicity and proliferation directed by a single chimeric TCRζ/CD28 receptor. Nat Biotechnol.

[CR52] Mashima Y, Kigasawa K, Wakakura M, Oguchi Y (2000). Do idebenone and vitamin therapy shorten the time to achieve visual recovery in Leber hereditary optic neuropathy?. J Neuroophthalmol.

[CR53] Master A (2019) Kymriah vs. Yescarta. Nucleus Biologics. https://nucleusbiologics.com/resources/kymriah-vs-yescarta/. Accessed 02 February 2020

[CR54] MedlinePlus (n.d.) Inborn errors of metabolism. MedlinePlus Medical Encyclopedia. https://medlineplus.gov/ency/article/002438.htm. Accessed 03 February 2020

[CR55] Mendes HF, Cheetham ME (2008). Pharmacological manipulation of gain-of-function and dominant-negative mechanisms in rhodopsin retinitis pigmentosa. Hum Mol Genet.

[CR56] MeSH (2020) "MeSH Descriptor Data: Metabolic diseases". National Library of Medicine. https://meshb.nlm.nih.gov/record/ui?ui=D008659. Accessed 03 February 2020

[CR57] MIT NEWDIGS Initiative (2017) Finances of Cures in the US (FoCUS) Research brief. Massachussetes Institute of Technology. https://newdigs.mit.edu/sites/default/files/FoCUS_Research_Brief_2017F211v011.pdf. Accessed 29 May 2020.

[CR58] Mitha F (2019) The return of Gene Therapy. Labiotech. https://www.labiotech.eu/features/gene-therapy-history/. Accessed 03 February 2020

[CR59] Naldini L, Blömer U, Gallay P, Ory D, Mulligan R, Gage FH (1996). In vivo gene delivery and stable transduction of nondividing cells by a lentiviral vector. Science..

[CR60] National Academy of Science (2017) Human Genome Editing - Science, Ethics and governance. National Academy of Science & National Academy of Medicine 28796468

[CR61] National Cancer Institute (2015) What Is Cancer?. National Cancer Institute: https://www.cancer.gov/about-cancer/understanding/what-is-cancer. Accessed 03 February 2020

[CR62] Novartis (2020) Understanding Kymriah. Kymriah: https://www.us.kymriah.com/acute-lymphoblastic-leukemia-children/about-kymriah/understanding-kymriah/

[CR63] Nuffield Council on Bioethics (2018). Patient access to experimental treatment.

[CR64] Patrício MI, Barnard AR, Xue K, MacLaren RE (2018). Choroideremia: molecular mechanisms and development of AAV gene therapy. Expert Opin Biol Ther.

[CR65] Psatha N, Reik A, Phelps S, Zhou Y, Dalas D, Yannaki E, Levasseur DN, Urnov FD, Holmes MC, Papayannopoulou T (2018) Disruption of the BCL11A Erythroid Enhancer Reactivates Fetal Hemoglobin in Erythroid Cells of Patients with β-Thalassemia Major. Mol Ther - Methods & Clinical Development 10:313–326 10.1016/j.omtm.2018.08.003 PMC6120587 30182035

[CR66] Raper SE, Chirmule N, Lee FS, Wivel NA, Bagg A, Gao GP, Wilson JM, Batshaw ML (2003). Fatal systemic inflammatory response syndrome in a ornithine transcarbamylase deficient patient following adenoviral gene transfer. Mol Genet Metab.

[CR67] Raty J, Pikkarainen J, Wirth T, Yla-Herttuala S (2008) Gene Therapy: The First Approved Gene-Based Medicines, Molecular Mechanisms and Clinical Indications. Curr Mol Pharmacol 1(1):13–23 10.2174/1874467210801010013 20021420

[CR68] Rochi A, Ronchi AM (2009). eCulture: cultural content in the digital age.

[CR69] Roselli EA, Mezzadra R, Frittoli MC, Maruggi G, Biral E, Mavilio F, Mastropietro F, Amato A, Tonon G, Refaldi C, Cappellini MD, Andreani M, Lucarelli G, Roncarolo MG, Marktel S, Ferrari G (2010) Correction of β‐thalassemia major by gene transfer in haematopoietic progenitors of pediatric patients. EMBO Mol Med 2(8):315–328 10.1002/emmm.201000083 PMC3377331 20665635

[CR70] Rosenberg SA, Aebersold P, Cornetta K, Kasid A, Morgan RA, Moen R, Karson EM, Lotze MT, Yang JC, Topalian SL, Merino MJ, Culver K, Miller AD, Blaese RM, Anderson WF (1990). Gene transfer into humans — immunotherapy of patients with advanced melanoma, using tumor-infiltrating lymphocytes modified by retroviral gene transduction. N Engl J Med.

[CR71] Tan YT, Ye L, Xie F, Beyer AI, Muench MO, Wang J, Chen Z, Liu H, Chen SJ, Kan YW (2018). Respecifying human iPSC-derived blood cells into highly engraftable hematopoietic stem and progenitor cells with a single factor. Proc Natl Acad Sci U S A.

[CR72] Terheggen HG, Lowenthal A, Lavinha F, Colombo JP, Rogers S (1975). Unsuccessful trial of gene replacement in arginase deficiency. Z Kinderheilkd.

[CR73] Ura T, Okuda K, Shimada M (2014). Developments in viral vector-based vaccines. Vaccines..

[CR74] What is Genome Editing? NIH (2019) Genome.gov: https://www.genome.gov/about-genomics/policy-issues/what-is-Genome-Editing. Accessed 03 February 2020

[CR75] WHO (2020) Genetic research. World Health Organization: https://www.who.int/genomics/research/en/. Accessed 15 February 2020

[CR76] Zafar SY, Abernethy AP (2013) Financial toxicity, Part I: a new name for a growing problem. Oncology (Williston Park) PMC4523887 23530397

[CR77] Zhang C, Oberoi P, Oelsner S, Waldmann A, Lindner A, Tonn T, Wels WS (2017) Chimeric antigen receptor-engineered NK-92 cells: an off-the-shelf cellular therapeutic for targeted elimination of cancer cells and induction of protective antitumor immunity. Front Immunol 8. 10.3389/fimmu.2017.00533 10.3389/fimmu.2017.00533 PMC5435757 28572802

